# Safely reopening and operating a primary healthcare facility after closure due to SARS-CoV-2 infection in a healthcare worker – Nairobi, Kenya, 2020

**Published:** 2024-10-28

**Authors:** Linus K Ndegwa, Daniel Kimani, Mercy Njeru, Tai-Ho Chen, Catherine Macharia, Annalice Ouma, Frankline O. Mboya, Julius Oliech, Titus K Kwambai, Ahmed Liban, Immaculate Mutisya, Rebecca Wangusi, Marc Bulterys, Taraz Samandari

**Affiliations:** 1 US Centers for Disease Control and Prevention, Nairobi, Kenya; 2 Nairobi County Department of Health, Nairobi, Kenya; 3 PACT Endeleza, Center for International Health, Education, and Biosecurity (CIHEB), Institute of Human Virology, University of Maryland School of Medicine, Baltimore, MD, USA

**Keywords:** infection prevention and control, healthcare worker, primary healthcare facility, COVID-19, SARS-CoV-2

## Abstract

The first COVID-19 case in a healthcare worker in Kenya was reported on March 30, 2020, in Nairobi, leading to a 41-day closure of the health facility where he had worked. We assessed infection prevention and control (IPC) activities and implemented recommendations to re-open and operate the facility. We conducted a risk assessment of the facility in April 2020 using a modified World Health Organization, six-element IPC facility risk assessment tool. IPC recommendations were made, and a follow-up assessment of their implementation was conducted in July 2020. Breaches in IPC measures included poor ventilation in most service delivery areas; lack of physical distancing between patients; inadequate COVID-19 information, education, and communication materials; lack of standard operating procedures on cleaning and disinfecting high-touch areas; insufficient IPC training; inadequate hand hygiene facilities; insufficient personal protective equipment supplies; and an inactive IPC committee. Strengthening IPC measures is critical to prevent healthcare facility closures.

Patients with unrecognized Severe acute respiratory syndrome coronavirus 2 (SARS-CoV-2) infection seeking care at primary healthcare facilities with inadequate infection prevention and control (IPC) measures can be a source of SARS-CoV-2 transmission (1). The first reported case of COVID-19 in Kenya was diagnosed in a traveler on March 13, 2020, and the first known infected healthcare worker (HCW) who had not traveled outside Kenya was identified on March 30, 2020, in a primary health facility in Nairobi. At the time, there was a high degree of uncertainty and concern about the novel pathogenic virus, as well as alarm over the well-being of frontline HCWs who had already been identified as being at high risk of infection and among whom had been some highly publicized deaths elsewhere in the world. The additional concern that HCWs may be a source of onward transmission to patients or their fellow HCWs, led to the closure of the Nairobi facility. We detail here the experiences of the events leading to the closure of this health facility following the identification of this case, potential exposure of others at the clinic and the process of safely reopening the facility.

Healthcare worker 1 (HCW-1) in the Outpatient Department attended to a patient on March 16, 2020. The patient had travelled from Dubai seven days earlier and had COVID-19 like symptoms, including cough, a runny nose, headache, and body aches. HCW-1 used gloves and a medical mask while examining the patient and subsequently referred the COVID-19 suspect patient to a designated COVID-19 treatment center on the same day per the Kenyan Ministry of Health (MoH) protocol. At the designated COVID-19 treatment center, although tests were available, the patient was not tested for COVID-19 but was given ibuprofen and instructed to self-isolate. On March 27, 2020, HCW-1 who was recently diagnosed with diabetes which is a risk factor for serious complications with COVID-19 (2) developed a cough and fever. This raised the level of urgency to know the SARS-CoV-2 infection status of the patient who had travelled from Dubai. On March 30, 2020, a nasopharyngeal sample was taken from HCW-1 and tested positive for SARS-CoV-2 by real-time reverse transcription polymerase chain reaction (rRT-PCR). HCW-1 was then admitted to a designated COVID-19 treatment center for management. The Nairobi primary healthcare facility was closed on March 30, 2020 for cleaning and fumigation and all services at the facility were closed to the public; the methadone assisted therapy (MAT) clinic remained opened only to dispense methadone to all its clients with services including counselling sessions, group therapy, and consultations being suspended. Given the shortage of SARS-CoV-2 test kits at the time, no outbreak investigation was conducted among patients who had attended the facility, and HCWs were instructed to self-quarantine for 14 days, after which they were tested for SARS-CoV-2 by rRT-PCR.

The disruption in essential services at the facility because of the COVID-19 pandemic was experienced by other facilities across the country as COVID-19 continued its anticipated trajectory and HCWs were at high risk of exposure (3). We report an overview of our experience and response in the disruption of healthcare services in this mid-sized primary healthcare facility in Kenya following the infection of HCW-1. We detail the steps taken to assess and implement recommendations to safely re-open the facility after closure because of SARS-CoV-2 infection in the HCW.

## Methods

### Setting

This primary healthcare facility has a bed capacity of 16, and 61 staff attending daily to 250–300 outpatients, 15–18 inpatient maternity cases, and 500–600 MAT clients. The facility is run by the Nairobi County Department of Health, and does not receive complicated cases or ambulances. The facility provides mostly drop-in primary healthcare services within Nairobi County including outpatients, uncomplicated maternity inpatients, and a specialized MAT clinic for an underserved, inner-city population. There are two entrances to the facility: one for MAT clients and another for other patients. Its laboratory is basic, conducting such tests as hemoglobin, urinalysis, and testing stool for ova and cysts.

### IPC assessments and recommendations

In April 2020, an IPC risk assessment was requested by the Kenya Ministry of Health (MoH) after the Nairobi County Department of Health ordered the closure of the primary healthcare facility. The MoH and the US Centers for Disease Control and Prevention (CDC) used a 26-item modified tool (4) from the World Health Organization (WHO) to identify the gaps in IPC. The WHO tool covers several key areas including: the IPC program, guidelines, and standard operating procedures (SOPs), training and monitoring, screening, triage, early recognition of persons with symptoms of COVID-19 and testing for SARS-CoV-2, the built environment, infrastructure and supplies, management of visitors, and maintenance of IPC interventions. We modified the tool to include injection safety and laboratory components.

**Fig. 1.**
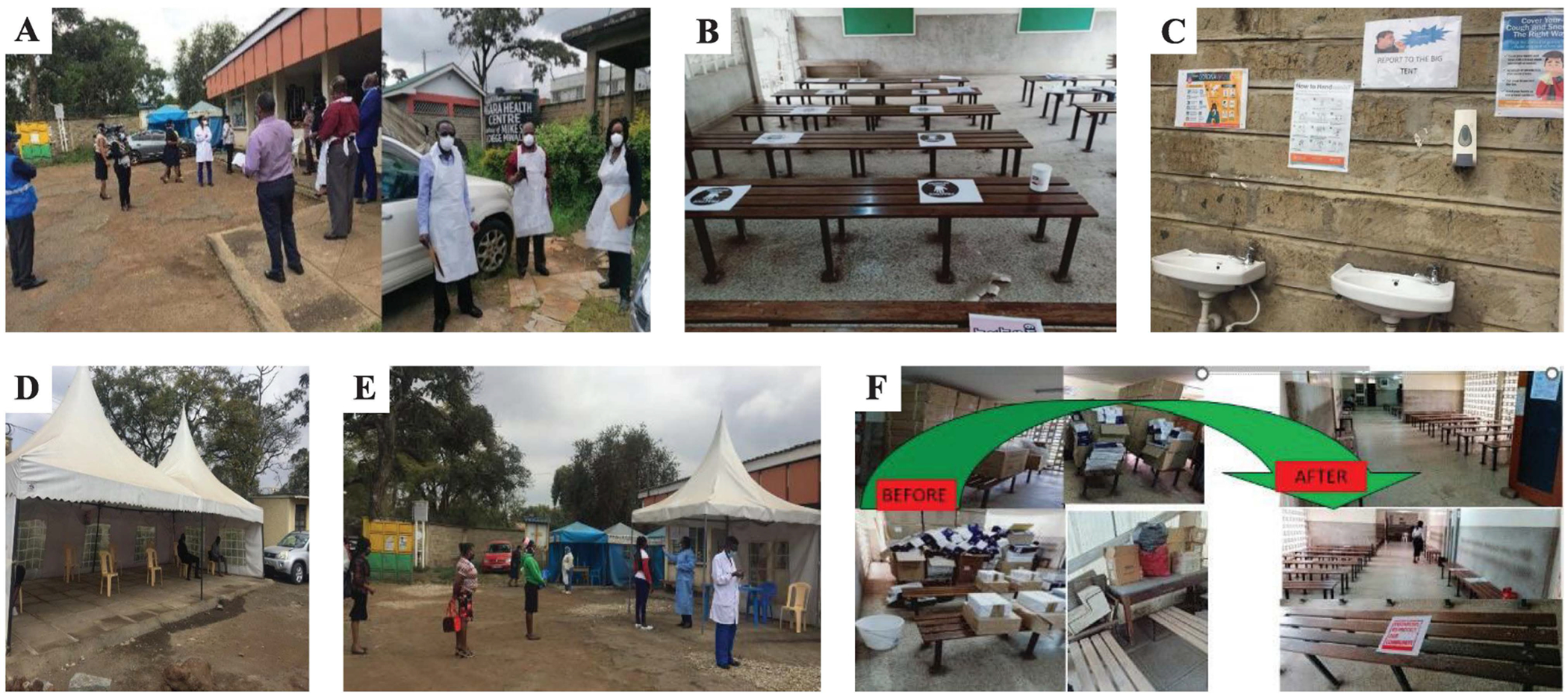
Triage area, respiratory and non-respiratory waiting bays with marked physical distancing at the Nairobi primary healthcare facility. A. Risk assessment team at the healthcare facility, B. Physical distancing markers at the non-respiratory patient waiting bay, C. Hand hygiene stations for clients with posted information, education, communication material, D. Open waiting bay for respiratory patients, E. Triage area, F. Improved ventilation at the waiting bays following assessment and removal of expired commodities.

Assessment teams included staff members from the Nairobi County Department of Health, University of Maryland (CDC implementing partner), and CDC (Figure 1A). Upon arrival at the facility, the assessment team first met with the facility’s officer-in-charge and the subcounty medical director to explain the assessment and obtain agreement to engage in the activity. After provision of their verbal consent, a paper-based semi-structured questionnaire (Supplementary Material) was administered to the facility’s officer-in-charge who is a clinician, the laboratory technician, and a support staff member who serves in a variety of non-technical roles. Other HCW were not interviewed. In addition, a paper-based questionnaire was administered and captured examination of the facility and observation of IPC practices focusing on patients/client’s workflows and the facility’s capacity in each of the IPC domains to prevent SARS-CoV-2 transmission. Based on the findings of the April assessment, recommendations were made, and a follow-up assessment was conducted on July 22, 2020, to appraise the progress of the implementation of the recommendations.

### Ethical concerns

This investigation was carried out by the Kenya MoH in collaboration with the Nairobi City County Health Department in response to the COVID-19 pandemic as authorized by the circular MoH/ADM/IPC/02/Vol. 1, July 21, 2020 which is based upon the Public Health Act (Prevention, Control and Suppression of Covid-19) Regulations, 2020 (L.N. No. 49 of 2020). The surveillance protocol was reviewed by University of Washington Human Subjects Division and the Ethical Review Committee of AMREF (ESRC P967/2021). This activity was also reviewed by CDC and was conducted consistent with applicable federal law and CDC policy.^1^

## Results

The results of the single day assessment indicated that IPC preparedness was deficient in key areas of the six domains (Table 1). Ventilation was noted to be poor in most service delivery areas, especially at the waiting bay, and was hindered by stocks of expired commodities (Figure 1F), as well as the lack of physical distancing of the patients and clients when being screened (Figure 1E). Information education and communication (IEC) materials (Figure 1C), related to COVID-19 prevention were lacking in strategic areas such as the patient waiting areas. Proper IPC practices were hindered by a lack of SOPs especially on proper mixing of solutions for cleaning and decontamination of the high touch areas; IPC training was also inadequate. The facility was affected by shortages of critical IPC components including hand hygiene (HH) stations and personal protective equipment (PPE) supplies including N95 respirators (not available), eye protection (goggles/shield), gowns, and gloves. The IPC committee, which is expected to champion IPC practices, was inactive and did not know their roles and responsibilities. The team noted that the facility also had no HCWs occupational health and safety program which would have supported the risk assessment of the exposed HCWs before the facility closed. Other deviations from best practices included improper waste disposal and a lack of clear signage to direct clients and patients.

Following the initial assessment, a total of 27 recommendations were made to improve IPC practices at the health facility (Table 1). At the follow-up assessment, of the 27 recommendations given, 15 were fully and seven partially implemented (Table 1). Key corrective actions taken included: the establishment of a triage desk to separate patients with respiratory symptoms from others in a well-ventilated waiting bay, clear markings on the floors and seating to reinforce physical separation, the creation of a station for donning and doffing PPE and provision of PPE, the training and activation of the IPC committee, COVID-19-specific training for all staff, development and implementation of IPC SOPs and IEC materials, and improvements in workflow and access to limit interactions between patients and HCWs. The recommendations that were only partially implemented were beyond the control of the facility (i.e., commodity issues and staff shortages) and these needs were communicated by the officer-in-charge to the County Health Department. The staff completed 14 days of mandatory self-quarantine, and all 60 were tested for SARS-CoV-2 by rRT-PCR before re-opening the facility. This testing also helped identify two more cases among HCWs – (presumably infected from the community since they were tested at the end of the quarantine period, although by this time MoH had reported a total of 197 confirmed cases , with 130 of these imported ) – just before re-opening the facility. The decision to re-open the facility was made as a directive by the County Department of Health, once it felt that most of the recommendations were met and that the SOPs were being followed. Staff training helped to create confidence in following IPC procedures. The facility implemented policies and procedures following the national guidelines for COVID-19 management and workplace safety (5–7), which included conducting risk exposure assessments and contact tracing for HCWs, to avoid further closures of the facility.

## Discussion

By December 2020, there were 96,251 confirmed cases of COVID-19 in Kenya; 3,042 of these cases were in HCWs (8). Potential risks for increased SARS-CoV-2 transmission among staff at a health facility include inadequate triaging of suspected COVID-19 cases, inactive IPC committee, lack of IPC leadership, inadequate PPE supplies, and inadequate IPC training and implementation (9). Healthcare facilities are ideal settings for transmission of SARS-CoV-2 because transmission might occur between patients, from patients to HCWs and vice versa or between HCWs, or could be a site for superspreading event in which one person infects an unusually large number of secondary cases (10–12).

Had IPC measures been in place, the closure of this mid-sized healthcare facility may have been avoided and prevented the loss of essential healthcare to an underserved, inner-city population with repercussions beyond that of just treatment of patients with COVID-19. The negative impact of the COVID-19 pandemic and other major epidemics on TB/HIV services, maternal & child health services, immunization coverage and maternal mortality have been documented elsewhere (13). In this facility, expectant mothers, MAT clients and many drop-in outpatients lost their source of care during the closure period.

Good IPC practices are important to prevent infection in facilities among patients who may already be sick or potentially immunocompromised (such as the methadone clinic attendees) and to protect HCWs from illness. Aerosol-generating procedures in healthcare settings also increase HCW risk of acquiring infection (14). Patients with respiratory symptoms (coughing and sneezing) can transmit the virus by droplets or aerosols (15) and overcrowding in health facilities limits the possibility of physical distancing.

To monitor potential SARS-CoV-2 infection and risk of other infectious diseases in healthcare facilities and provide actionable data to reduce transmission, assessments are an essential component of IPC programs. The WHO recommends that national IPC programs should adopt validated risk assessment tools that fit their contexts (9). IPC gaps can be identified and remedied during periodic facility assessment before or after HCW exposures to SARS-CoV-2 (15). However, during a pandemic caused by a novel pathogen such as SARS-CoV-2, preexisting assessment tools can be adopted and modified as the scientific knowledge expands. We used the WHO infection control assessment tool (ICAT) (16) and a MoH an IPC healthcare facility response for COVID-19 guidance which we modified.

The WHO has outlined recommendations that can be used to reduce the risk of SARS-CoV-2 transmission in healthcare settings (17). Screening and triaging of every patient entering a healthcare facility for signs and symptoms of COVID-19 at the first entry point to the facility is paramount. After our assessment, a registration and triage desk were put in place at the facility to ensure that all patients are screened and isolated to reduce risk of transmission to other patients and HCWs.

Optimizing the use of engineering controls to reduce or eliminate exposures by shielding HCW and other patients from infected individuals is critical (14). Such measures can be inexpensive; for example, in this facility outdoor waiting bays and triage stations for patients and an outdoor facing pharmacy to dispense medications were well-implemented. The facility implemented an electronic medical record (EMR) system to identify patient–provider interactions and to facilitate contact tracing.

Healthcare delivery requires close physical contact between patients and HCWs. However, when possible, physical distancing (i.e., maintaining 1.5 meters between patients) is an important strategy to prevent SARS-CoV-2 transmission (14). The facility reorganized the waiting rooms so patients could sit at least 1.5 m apart. This was achieved by labeling sitting areas (Figure 1B) and incorporating additional space outside (Figure 1D). It would be preferable to have fewer staff per shift; however, this was not possible because of staff shortages that existed prior to the COVID-19 pandemic (18).

Appropriate use of PPE reduces the risk of exposure to respiratory secretions during patient care (14). The global shortage of PPE at the time of the pandemic required rational and appropriate use of PPE (19). At the time of the initial assessment, there was an inadequate supply of PPE at the facility, but this was resolved at the time of the follow-up visit. Nevertheless, Five of the recommendations were not implemented by the time of follow-up because they required more time to ensure full implementation.

Access to healthcare was compromised even after reopening with fewer (50–100) daily outpatient department visits compared with 250–300 client’s pre-pandemic seeking services at the facility (20). This could have been because of stigma directed at the facility after it had been publicized that there had been a case of COVID-19 in one of the HCW and also because fewer staff were available to offer services because they were in quarantine or isolation.

The IPC infrastructure findings in this facility were similar to reports from many other facilities in Kenya (21–23). Although these findings were early in the pandemic, their relevance remains. Most of the non-pharmaceutical measures recommended then such as IPC program improvements, HH, ventilation, physical distancing, cough etiquette, triaging of patients, occupational health and safety programs for HCWs are still relevant to prevent transmission of SARS-CoV-2 and any other infectious diseases. Even with the wide availability of COVID-19 vaccines, IPC measures remain important. The recommendations for this facility remain relevant as the knowledge of SARS-CoV-2 transmission has evolved.

The findings in this report are subject to some limitations. Firstly, we were not able to directly observe all the staff of the facilities and observe their IPC practices. However, we interviewed a small number of staff who were preparing the facility for re-opening. We have no reason to believe the staff interviewed were different from those not interviewed. Secondly, the assessment was done when the actual patients were not present. Instead, we relied on interviewed staff explanations which might limit the interpretation of some assessment areas such as client flow and waiting areas. However, we noted no major deficiencies during the later IPC assessment.

## Conclusion

Response to the COVID-19 pandemic has necessitated rapid scale-up and implementation of IPC measures at primary healthcare facilities where the risk for encountering patients with COVID-19 increases with higher rates of community transmission. Effective infection prevention and control readiness at primary healthcare facilities is vital in preventing the spread of infectious disease and propagation of a pandemic. If health facilities are closed because of disease outbreaks, community sensitization efforts may be important to reduce stigma and raise confidence in the safe resumption of services at the health facility.

## Supplementary Material

Supplement 1

Supplement 2

## Figures and Tables

**Table 1.** Infection prevention and control findings, recommendations, and follow-up in a primary healthcare facility that had closed after a healthcare worker COVID-19 case in Nairobi, Kenya, 2020

Key findings (April 2020)	Recommendations (April 2020)	Follow-up (July 2020)
**Infrastructure and triage**		
The standard waiting area did not separate patients with suspected COVID-19	Set up a triage area for incoming registration and waiting bay for suspected COVID-19 case	• A registration and triaging desk for adults and children with clear signage^‡^ • A separate waiting bay for suspected COVID-19 case in a large open tent were created (Figure 1)^‡^ • Staff at the triage areas were provided with face shield^‡^
No physical distancing guidance	Provide markings for physical distancing	• Clear markings were painted on the floor and stickers put on the seats to ensure physical distance of 1.5 m^‡^ • Posters emphasizing physical distancing were placed in strategic locations^‡^
No station for staff donning and doffing of personal protective equipment (PPE)	Provide staff donning/doffing station	One of the rooms was converted to a donning and doffing station and shelves were put in place for the PPE materials^‡^
Various items were stored in the corridors obstructing ventilation	Remove items obstructing ventilation	Items that were obstructing ventilation were removed and kept in a storage room^‡^
The waiting areas are small, and not able to accommodate large number of patients while maintaining one-meter physical distance	Limit patients in the internal waiting areas by opening outdoor waiting bays	• The open compound was cleared to allow more waiting space^‡^ • An open tent was installed as an overflow waiting bay^‡^
Insufficient hand washing and hand sanitizer stations	Provide additional hand hygiene stations	Running water and soap available; multiple hand washing, and sanitizing stations were put in strategic locations with job aids on how to perform proper hand hygiene^‡^
Inadequate lighting and ventilation in some of the rooms, especially in the methadone assisted therapy (MAT) clinic	Improve lighting and ventilation in the data room and triage of the MAT clinic	Additional lightbulbs were put in place to allow more lighting; however, windows were still not open because of pungent smell from outside^‡^
Pungent smell external to the facility	In consultation with county government remove the items producing smell near the facility	The items producing pungent smell not removed*
**Infection Prevention and Control (IPC) Program**		
The facility IPC committee was inactive	Activate IPC committee with regular meeting times	IPC committee has been activated with regular meeting schedules and clear terms of reference including minutes of meetings^‡^
The MAT clinic did not have an IPC committee	Consider having a separate IPC, committee for MAT clinic since they have a large staff (20)	An IPC committee was set up for the MAT clinic^‡^
Very few staff had received IPC and COVID-19 specific training	Train all staff on COVID-19 IPC, including cleaners and ambulance drivers	All staff trained^‡^
No updated standard operating procedures (SOPs) or job aids on various IPC-related procedures	Develop and adapt national SOPs, job aids on IPC and COVID-19 prevention	SOPs, job aids, posters were developed and adapted and positioned in strategic areas^‡^
**PPE**		
The facility had an insufficient supply of PPE	Provide PPE and ensure rational use as per Ministry of Health guidance	Adequate supply of PPE was provided, and staff trained on rational use^‡^
Not all MAT clients were wearing masks at the facility	Provide masks for MAT and other clients who visit the facility without masks.	The facility has insufficient masks for the MAT clients^‡^
MAT clients were too close to the pharmacist at the dispensing window	Provide face shields to staff working in the MAT pharmacy	Face shields were used by the MAT pharmacy staffs^‡^
**Cleaning and disinfection**		
High touch areas cleaned once a day or not at all.	Increase the frequency of cleaning and disinfection for high touch surfaces to three times a day with clear documentation	Frequency was increased but no documentation for confirmation and accountability*
**Occupational safety and health (OSH)**		
Normal standard work shifts were in operation except in the MAT clinic which had modified their work schedules	The staff to re-organize work shifts in cohorts in case of COVID-19 exposure so that a cohort could quarantine without service interruption	This has been partially achieved in some departments including maternity^†^
No occupational health and safety program	Establish an occupational health and safety program based on the ‘Ministry of Health Guidelines of OSH programs in healthcare settings’	The establishment of occupational health and safety program is ongoing*
No consideration of healthcare worker (HCW) comorbidities status in workstation placement	Conduct assessment of HCW for comorbidities to inform work placement	This has not been achieved because of staff shortage*
No clear system to follow-up HCWs who had been exposed to COVID-19	• Setup systems by following the national guidelines for COVID-19 exposures for HCWs • Testing of all the HCWs before resuming work	• Systems have been set up through orienting HCWs, conducting risk exposure assessment and following up of HCWs^†^ • Two more HCWs tested positive for COVID-19
Not all HCWs had received complete hepatitis B virus vaccine series	Provide hepatitis B virus vaccine for all HCWs	By July 2020, 33% of the facility HCWs were vaccinated through the Nairobi Metropolitan Service in collaboration with the Kenya Expanded Program on Immunization^†^
No psychosocial support for HCWs	Provide psychosocial support and counselling to the HCWs	Psychosocial support was only received during the isolation and quarantine period^†^
**Workflow: Maternal and Child Health (MCH), laboratory, and other areas**		
There was overcrowding in various departments with inadequate ventilation	Re-design the workflow in the facility to reduce congestion in enclosed spaces.	This was partially implemented with some congestion noted at the MCH waiting area^†^
Difficult to trace patient movement–Health worker contacts and flow in the facility	Introduce an electronic medical record (EMR) system to be used with the re-organized workflow	EMR installed, staff trained on how to use it and can clearly follow which clients interacted with which staff^‡^
There was no access control to the laboratory.	Re-design the laboratory area to set up access control.	Access control was set up through innovative redesigning of the laboratory space^‡^
There were no educational materials for COVID-19 at the patient waiting areas	Provide information, education, communication material on COVID-19 prevention at all patient waiting areas	Information, education, communication materials on COVID-19 prevention have been availed and placed in all the patient waiting areas^‡^
The laboratory phlebotomy area had a wooden table which is difficult to clean when contaminated	Non-absorbent phlebotomy table recommended.	A non-absorbent material was improvised to cover the table^†^
There were no written protocols for transfer of a confirmed or suspected COVID-19 in a patient	Develop clear protocols on transfer of confirmed or suspected COVID-19 in a patient to designated COVID-19 treatment facility	The transfer protocols are not yet documented*

* Not implemented;

† Partially implemented;

‡ Fully implemented.

## Footnotes

1 See e.g., 45 C.F.R. part 46. 102(1)(2), 21 C.F.R. part 56; 42 U.S.C. §241 (d); 5 U.S.C. §552a; 44 U.S.C. §3501 et seq.
