# Supplementary material for: Safely reopening and operating a primary healthcare facility after closure due to SARS-CoV-2 infection in a healthcare worker – Nairobi, Kenya, 2020
Source: Int J Infect Control. Author manuscript; Available in PMC 2025 Jul 1. (PMC12212145)
Supplement: Supplement 1 [file NIHMS2080325-supplement-Supplement_1.pdf]

IPC HEALTH FACILITY RISK ASSESSMENT TOOL IN CONTEXT OF COVID 19

**FACILITY DEMOGRAPHICS**				
Facility Name				
County	Sub-county			
Ownership of facility	☐ Public Health ☐ Faith Based ☐ Private ☐ Academic hospital Academic hospital			
MFL code				
Date of Assessment				
Rationale for Assessment	☐ Outbreak		☐ Routine	☐ Other (specify):
List available services/departments here				
List type and number of health workers here:				
What is the average daily number of outpatients in your facility?				
What is the average daily number of inpatients in your facility?				
Bed capacity	Total Beds___________Adult beds____________newborn beds_________ Bed occupancy			
Are family caretakers allowed to stay overnight in the patient care areas in adult wards?	☐Yes	☐No		

**1.**	**INFECTION CONTROL PROGRAM**			
	**Elements to be assessed**	**Assessment**		**Notes/Areas for Improvement**
a.	Is the 2015 national IPC policy available?	☐Yes	☐No	
b.	Is the 2015 national IPC guideline available?	☐Yes	☐No	
c.	Do you have facility-specific general IPC policies that are regularly updated?	☐Yes	☐No	
d.	Do you have facility-specific general IPC SOPs that are regularly updated?	☐Yes	☐No	
e.	Do you have TB-specific IPC policy?	☐Yes	☐No	
f.	Do you have TB-specific IPC plan?	☐Yes	☐No	
g.	Do you have an IPC committee?	☐Yes	☐No	
h.	Is there financial support available for Infection Prevention and Control program activities?	☐Yes	☐No	
i.	Is there a person or team of people responsible for conducting Infection Prevention and Control activities in your facility?	☐Yes	☐No	
j.	List activities for IPC committees:			
**2.**	**INFECTION CONTROL TRAINING AND COMPETENCY**			
	**Elements to be assessed**	**Assessment**		**Notes/Areas for Improvement**
a.	Is there a structured curriculum / training module for Infection Control	**Yes**	**No**	
b.	How many HCW have been trained on IPC in this facility?			
c.	Have you had a refresher training?	**Yes**	**No**	
d.	When was the last refresher training?			
e.	How many HCW have had COVID-19 specific IPC training?			
f.	List by the cadres:			

3.	HEALTHCARE WORKER SAFETY			
	**Elements to be assessed**	**Assessment**		**Notes/Areas for Improvement**
a.	Facility has an exposure control plan that is tailored to the specific requirements of the facility (e.g., addresses potential hazards posed by specific services provided by the facility).	☐Yes	☐No	
b.	HCW for whom contact with blood or other potentially infectious material is anticipated are trained on IPC upon hire and at least annually.	☐Yes	☐No	
c.	Following an exposure event, post-exposure evaluation and follow-up, including prophylaxis (PEP) as appropriate, are available at no cost to employee and are supervised by a licensed healthcare professional.	☐Yes	☐No	
d.	Facility tracks HCW exposure events and evaluates event data and develops/implements corrective action plans to reduce incidence of such events.	☐Yes	☐No	
e.	Facility offers Hepatitis B vaccination for its workers	☐Yes	☐No	
f.	All HCW receive baseline tuberculosis (TB) screening prior to placement; HCW receive repeat testing, if appropriate, based on the facility-level risk assessment.	☐Yes	☐No	
g.	Facility has well-defined policies concerning contact of personnel with patients when personnel have potentially transmissible conditions. These policies include:	☐Yes	☐No	
i.	Work-exclusion policies that encourage reporting of illnesses and do not penalize with loss of wages, benefits, or job status.	☐Yes	☐No	
ii.	Is there a focal person or (team of persons) responsible for managing occupational safety and health activities in your hospital			
iii.	Education of personnel on prompt reporting of illness to supervisor.	☐Yes	☐No	
h.	Do you have a policy for routine evaluation to probable exposures e.g. to COVID-19	☐Yes	☐No	
i.	Do you have a policy on the management and follow-up of a HCW with confirmed exposure to COVID-19?	☐Yes	☐No	
j.	Number of hand hygiene stations per department:			
k.	Supplies necessary for adherence to hand hygiene are readily accessible to HCW in patient care areas.	☐Yes	☐No	
l.	Soap	☐Yes	☐No	
m.	Running Water	☐Yes	☐No	
n.	Paper towels	☐Yes	☐No	
o.	Alcohol-based hand rub (Sanitizer)	☐Yes	☐No	

4. TRIAGE				
	**During infectious disease outbreaks, triage is particularly important to separate patients likely to be infected with the respiratory pathogen of concern.**			**Comments and Notes**
1.	Visual alerts (signage) are posted at the entrance of the facility and in strategic areas to guide patients with respiratory symptoms to immediately report to the registration desk patients	Yes	No	
2.	Routine clinical triage already in place in healthcare facility (Modification in respiratory outbreak-increased ventilation)	Yes	No	
3.	Triage SOP is developed in the context of the COVID-19 and other infectious diseases	Yes	No	
4.	The facility has a separate registration desk for patients coming in with respiratory symptoms	Yes	No	
5.	Staff are trained and updated on triage procedures	Yes	No	
6.	A register/document to capture all those visiting the facility (Both patients and non-patients AND staff)<br>Details captured in the records:	Yes	No	
a.	Name__	Yes	No	
b.	Phone Contact___	Yes	No	
c.	Place of Residence____	Yes	No	
d.	Sub-county	Yes	No	
7.	**The following are available at registration desk:**			
a.	Facemasks (Or patients have own mask)	Yes	No	
b.	Adequate hand hygiene stations (water and soap)	Yes	No	
c.	Paper towels/ disposable	Yes	No	
d.	A waste bin with lid	Yes	No	
e.	Bin liners			
f.	Hand washing area signages	Yes	No	
g.	Cough Etiquette protocols and signage	Yes	No	
h.	COVID -19 Informational Signages	Yes	No	
i.	Directional signages on patient triage	Yes	No	
j.	HCW has appropriate PPE at the triage area	Yes	No	
8.	**Patient waiting areas**			
a.	Hand hygiene stations available	Yes	No	
b.	Hand hygiene adequate in waiting areas	Yes	No	
9.	Patients are organized in a way that they maintain appropriate distance	Yes	No	
10.	Administrative staff in the facility are trained on the triage procedures	Yes	No	

a.	Total administrative staff: ________ Total Trained: ___________			
11.	Administrative staff have the appropriate PPE for their triage activities	Yes	No	
a.	List the available PPE: Gloves______ Surgical masks_______ N95: _________ Gown: _______			

5. PERSONAL PROTECTIVE EQUIPMENT				
	**Elements to be assessed**	**Assessment**		**Notes/Areas for Improvement**
1.	**Adequate and appropriate PPE is available and readily accessible to HCW**			
a.	Where do you don and doff your PPE including respirators			
b.	Hand hygiene is performed immediately after removal of PPE.	Yes	No	
c.	Overall observation including hand hygiene facilities:	Yes	No	
2.	**Gloves**			
a.	HCW wear gloves for potential contact with blood, body fluids, mucous membranes, non-intact skin, or contaminated equipment.	Yes	No	
b.	Do not wear the same pair of gloves for the care of more than one patient.	Yes	No	
c.	Do not wash gloves for the purpose of reuse.	Yes	No	
d.	Overall observation:			
3.	**Gowns**			
a.	HCW wear gowns to protect skin and clothing during procedures or activities where contact with blood or body fluids is anticipated.	Yes	No	
b.	Overall observation:			
4.	**Masks**			
a.	HCW wear N95 masks when handling patients with respiratory symptoms or when carrying out procedure with possible aerosol generation	Yes	No	
b.	Surgical Masks available for use by the health care workers as required	Yes	No	
c.	Overall observation:			
5.	**Facial protection**			
a.	HCW wear mouth, nose, and eye protection during procedures that are likely to generate splashes or sprays of blood or other body fluids.	Yes	No	
b.	Overall observation:			

6. LABORATORY				
	**Elements to be assessed**	**Assessment**		**Notes/Areas for Improvement**
1.	Hand hygiene stations available and adequate in laboratory waiting area	Yes	NO	
2.	Cough Etiquette protocols and signage	YES	NO	
3.	COVID -19 Informational Signages	YES	NO	
4.	Laboratory staff are trained on COVID-19 IPC	YES	NO	
5.	Laboratory staff are trained on General Lab biosafety	YES	NO	
6.	Laboratory staff have adequate and appropriate PPE	YES	NO	
i.	List available PPE: PPE: Gloves_____ Surgical masks_______ N95: _________ Gown: _______			
7.	Hand washing sink with running water and soap/ alcohol-based hand rub	Yes	NO	
i.	Hand hygiene with alcohol-based hand rub	Yes	NO	
8.	The laboratory has IPC and biosafety procedures in use and up to date	Yes	NO	
a.	Safe specimen handling (decontamination of the specimen container)	YES	NO	
b.	Post Exposure Prophylaxis	YES	NO	
c.	Proper Donning and Doffing of PPE	YES	NO	
d.	Hand Hygiene procedure	YES	NO	
e.	Equipment disinfection procedures	YES	NO	
f.	Surface disinfection procedures and how frequent this is done (review checklist)	YES	NO	
g.	Waste disposal procedures	YES	NO	
**6.**	**Laboratory safety equipment**			
a.	Procedure on Proper use of BSC/TB Safety hood	Yes	NO	
b.	Aerosol generating procedures conducted in Safety hood or BSC	Yes	NO	
c.	BSC/TB Safety Hood has update certification	YES	NO	
d.	Proper ventilation in the laboratory area	YES	NO	
13	**Laboratory Waste Management**			
a.	SOP on waste management available in the lab	YES	NO	
b.	IEC material on waste segregation available and displayed	YES	NO	
c.	Waste treatment before the waste leave the laboratory	YES	NO	
i.	If yes: Autoclaving_____________Chemical Decontamination: ______________			

ii.	Explain the process of treatment____________________			
d	Final waste disposal facilities available at the site			
	Open burning_____ Incineration: _______ Outsourced Service:_____			

7. INJECTION SAFETY				
	**Elements to be assessed**	**Assessment**		**Notes/Areas for Improvement**
1.	**Injection safety: Review all areas handling injections and sharps in the facility**	Yes	NO	
2.	Injections are prepared using aseptic technique in a clean area free from contamination or contact with blood, body fluids or contaminated equipment.	YES	NO	
3.	Needles and syringes are used for only one patient (this includes manufactured prefilled syringes and cartridge devices such as insulin pens).	YES	NO	
4.	The rubber septum on a medication vial is disinfected with alcohol prior to piercing.	YES	NO	
5.	Medication containers are entered with a new needle and a new syringe, even when obtaining additional doses for the same patient.	YES	NO	
6.	Single dose (single-use) medication vials, ampules, and bags or bottles of intravenous solution are used for only one patient.	YES	NO	
7.	Medication administration tubing and connectors are used for only one patient.			
8.	Staff trained on Injection safety and safe phlebotomy	Yes	NO	
9.	All sharps are disposed of in a puncture-resistant sharps container.			
10.	Filled sharps containers are disposed of in accordance with state regulated medical waste rules.			
a	Protocols on waste segregation are available at all generation points	YES	NO	
b	IEC Materials on waste segregation available at all points	YES	NO	
c	Verify how final disposal of sharps is done	YES	NO	
d	Post Exposure Protocols are available at the site	YES	NO	
e	Post Exposure prophylaxis available at the site at all the times (Verify)	YES	NO	
	**8. TRANSPORTING PATIENTS TO TREATMENT OR ISOLATION CENTRE**			
1.	Is there any protocol for transfer of patients to isolation centre?	YES	NO	
2.	Is there separate ambulance available for transporting patients to isolation centre	YES	NO	
**3.**	Are the ambulance staff trained in wearing PPE & infection control practices?			
4.	How far is the Isolation facility from the health center	Yes	NO	

	9. ENVIRONMENTAL CLEANING			
11	Supplies necessary for appropriate cleaning and disinfection	YES	NO	
12	What do you have? Include %	YES	NO	
13	How to prepare High-touch surfaces in rooms where surgical or other invasive procedures (e.g., endoscopy, spinal injections) are performed are cleaned and then disinfected with an disinfectant after each procedure.			
a	Cleaners and disinfectants are used in accordance with manufacturer's instructions (e.g., dilution, storage, shelf-life, contact time).	YES	NO	
b	Do you have an SOP on preparation of disinfection solutions?	YES	NO	
c	HCW engaged in environmental cleaning wear appropriate PPE to prevent exposure to infectious agents or chemicals (PPE can include gloves, gowns, masks, and eye protection).	YES	NO	

Note: The exact type of correct PPE depends on infectious or chemical agent and anticipated type of exposure.

10. Device Reprocessing

	Elements to be assessed	Assessment		Notes/Areas for Improvement
1.	Policies, procedures, and manufacturer reprocessing instructions for reusable medical devices used in the facility are available in the reprocessing area(s).	Yes	NO	
2.				
3.	Devices are thoroughly cleaned according to manufacturer instructions* and visually inspected for residual soil prior to sterilization.	YES	NO	
4.	What reprocessing method is used?			
5.	Reusable medical devices are cleaned, reprocessed (disinfection or sterilization) and maintained according to the manufacturer instructions.	YES	NO	
6.	How do you confirm you have achieved sterility/disinfection?			
7.	Single-use devices are discarded after use and not used for more than one patient unless they have been appropriately reprocessed as described in the note below.	YES	NO	

8.	**Reprocessing area:**			
a.	Adequate space is allotted for reprocessing activities.	Yes	NO	
b.	A workflow pattern is followed such that devices clearly flow from high contamination areas to clean/sterile areas (i.e., there is clear separation between soiled and clean workspaces).	Yes	NO	
c.	Adequate time for reprocessing is allowed to ensure adherence to all steps recommended by the device manufacturer, including drying and proper storage.	Yes	NO	
d.	Do you have a stopwatch for this?	YES	NO	
Note: Facilities should have an adequate supply of instruments for the volume of procedures performed and should schedule procedures to allow sufficient time for all reprocessing steps.				
e.	HCW engaged in device reprocessing wear appropriate PPE to prevent exposure to infectious agents or chemicals (PPE can include gloves, gowns, masks, and eye protection).	YES	NO	
f.	Medical devices are stored in a manner to protect from damage and contamination.	YES	NO	
g.		YES	NO	

**Note: If the manufacturer does not provide such instructions, the device may not be suitable for multi-patient use*

Adapted from WHO reference number: WHO/2019-nCoV/HCF_assessment/IPC/2020.1
