# Supplementary figures and images for: Safely reopening and operating a primary healthcare facility after closure due to SARS-CoV-2 infection in a healthcare worker – Nairobi, Kenya, 2020
Source: Int J Infect Control. Author manuscript; Available in PMC 2025 Jul 1. (PMC12212145)

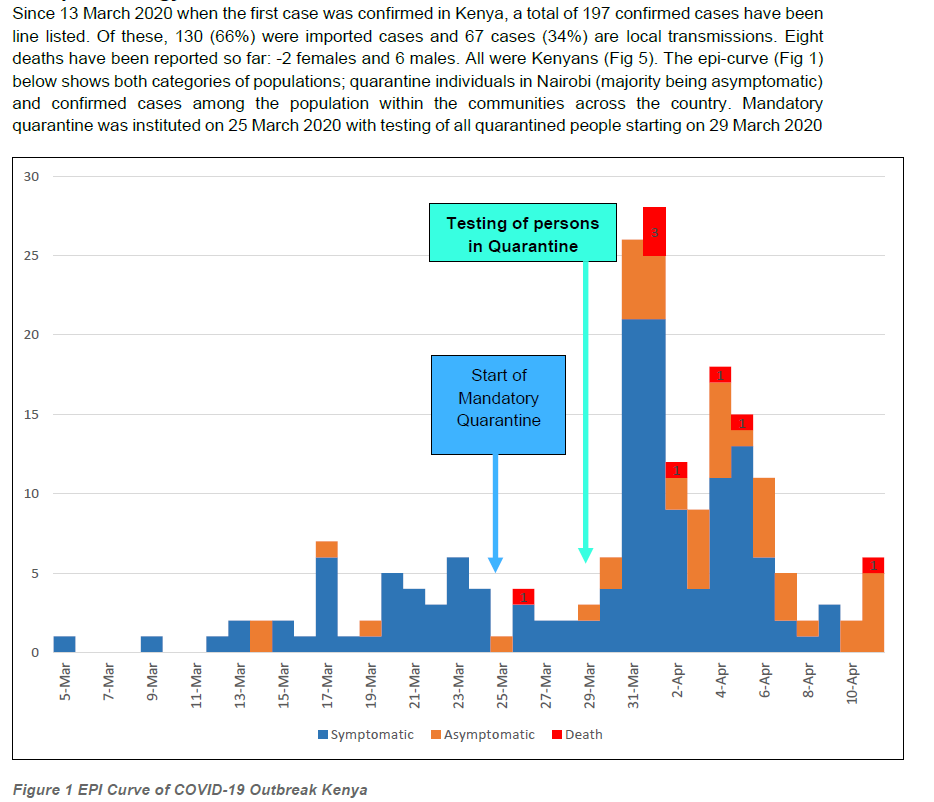

Supplement: Supplement 2 [file NIHMS2080325-supplement-Supplement_2.docx]
